# Automatic application of neural stimulation during wheelchair propulsion after SCI enhances recovery of upright sitting from destabilizing events

**DOI:** 10.1186/s12984-018-0362-2

**Published:** 2018-03-12

**Authors:** Kiley L. Armstrong, Lisa M. Lombardo, Kevin M. Foglyano, Musa L. Audu, Ronald J. Triolo

**Affiliations:** 1 Department of Biomedical Engineering, Case Western Reserve University, Cleveland, OH USA; 2 Advanced Platform Technology Center, Cleveland Louis Stokes Veterans Affairs Medical Center, 10701 East Boulevard, Cleveland, OH 44106 USA

**Keywords:** Spinal cord injury, Functional neuromuscular stimulation, Event detection

## Abstract

**Background:**

The leading cause of injury for manual wheelchair users are tips and falls caused by unexpected destabilizing events encountered during everyday activities. The purpose of this study was to determine the feasibility of automatically restoring seated stability to manual wheelchair users with spinal cord injury (SCI) via a threshold-based system to activate the hip and trunk muscles with electrical stimulation during potentially destabilizing events.

**Methods:**

We detected and classified potentially destabilizing sudden stops and turns with a wheelchair-mounted wireless inertial measurement unit (IMU), and then applied neural stimulation to activate the appropriate muscles to resist trunk movement and restore seated stability. After modeling and preliminary testing to determine the appropriate inertial signatures to discriminate between events and reliably trigger stimulation, the system was implemented and evaluated in real-time on manual wheelchair users with SCI. Three participants completed simulated collision events and four participants completed simulated rapid turns. Data were analyzed as a series of individual case studies with subjects acting as their own controls with and without the system active.

**Results:**

The controller achieved 93% accuracy in detecting collisions and right turns, and 100% accuracy in left turn detection. Two of the three subjects who participated in collision testing with stimulation experienced significantly decreased maximum anterior-posterior trunk angles (*p* < 0.05). Similar results were obtained with implanted and surface stimulation systems.

**Conclusions:**

This study demonstrates the feasibility of a neural stimulation control system based on simple inertial measurements to improve trunk stability and overall safety of people with spinal cord injuries during manual wheelchair propulsion. Further studies are required to determine clinical utility in real world situations and generalizability to the broader SCI or other population of manual or powered wheelchair users.

**Trial registration:**

ClinicalTrials.gov Identifier NCT01474148. Registered 11/08/2011 retrospectively registered.

## Background

There are approximately 285,000 people in the United States living with spinal cord injuries (SCI) [1], roughly two thirds of whom are dependent on scooters or wheelchairs for daily mobility [2]. According to a survey of individuals with SCI, trunk stability is among the top functions they desire to improve or restore [3]. People with SCI who have lost the ability to control their trunks experience more postural sway during sitting than able-bodied individuals [4]. Minor disturbances can destabilize upright sitting and cause a loss of erect sitting posture during wheelchair use, leading to injurious falls and thus limiting their mobility and independence. Tips and falls are the leading cause of injury for people in wheelchairs, accounting for two-thirds of the more than 100,000 wheelchair related injuries per year requiring treatment in emergency departments [5]. Wheelchair accidents can result in fractures, lacerations, contusions and abrasions, and even death [6]. Rehabilitation for a fracture in an individual with SCI requires four to eight weeks of hospital stay, resulting in reduced strength and increased risk of blood clots [6]. Additionally, cost of treatment due to wheelchair-related falls typically ranges from $25,000 to $75,000 [6]. The riding surface and environmental factors [7], wheelchair design (such as caster size and seat position) [8], and center of gravity location [9] can all affect wheelchair stability, and ultimately contribute to falls and tips experienced by manual wheelchair users. Common events that can contribute to destabilization include colliding with an obstacle (such as a low curb or wall), propelling through sharp turns, negotiating uneven or inclined surfaces, and maneuvering curb drops [7, 10].

Current methods to maintain trunk stability for manual wheelchair users with SCI include seat belts to keep the trunk from falling forward, seat cushioning systems to decrease the exaggerated posterior pelvic tilt that results from paralysis of the hips and trunk, increased seat dump to take advantage of gravity in a backwards leaning posture, or supports to limit lateral motions of the trunk. These methods have many disadvantages, including reduced work volumes and impaired ability to reach and manipulate objects due to the external constraints, as well as pressure ulcers, skin tears, lowered self-esteem and even asphyxiation [11]. Non-compliance with such strategies is high since they also restrict desired motions and interfere with functional tasks while the wheelchair is not moving [12]. Power wheelchairs offer more options for specialized seating systems and can provide power tilting and reclining to allow users to independently change their postures in preparation for a task or anticipated disturbance. However, they impose the same restrictions on voluntary motion, and people are typically reluctant to switch from manual to powered wheelchairs for the additional stability as it may be viewed as an indication of greater disability [13].

Functional neuromuscular stimulation (FNS) has been utilized to restore and maintain trunk stability by significantly increasing the trunk extension moment [14] and multidirectional trunk stiffness [15, 16]. When the trunk flexor and extensor muscles are activated with neural stimulation, people show physical and psychological improvements of strength and stability, particularly in response to unexpected, destabilizing forces [17, 18]. EMG of healthy individuals has shown significant activation of the erector spinae muscles in response to perturbations pulling the trunk anteriorly [19]. Continuous activation of the otherwise paralyzed hip and trunk muscles with FNS can maintain trunk stability during similar anteriorly-directed forces applied to the trunks of individuals with SCI [17, 20]. Furthermore, activating the hip and trunk muscles with neural stimulation can restore upright sitting from forward flexed or side-leaning positions automatically without use of the arms based on exceeding an angular threshold monitored by a tilt sensor attached to the sternum [21]. Similarly, a powered-wheelchair neuroprosthesis that activated trunk flexors and extensors with FNS on able-bodied participants experiencing perturbations in the anterior-posterior (AP) direction showed a decrease in trunk displacement and velocity with stimulation [18]. Additionally, continuous electrical stimulation of the trunk and hip extensors has been shown to improve the mechanics of manual wheelchair propulsion for people with SCI by decreasing the peak resultant pushrim forces and increasing propulsion efficiency [22]. These studies suggest that simple, automatic systems based on body position have the potential to improve mobility and voluntary function while sitting in a wheelchair, however automatically modulating stimulation at the onset of destabilizing events during manual wheelchair propulsion to minimize the effects of unanticipated perturbations or restore seated posture with neural stimulation has not yet been examined in person with SCI under simulated real-world conditions.

To employ neural stimulation in response to potentially destabilizing events, a method of accurately predicting or detecting such situations must be identified. Inertial measurement units (IMUs) have been utilized to monitor trunk angle of people with SCI in a static environment [21], determine phases of the manual wheelchair stroke cycle [23], and classify physical activities of daily living [24]. Additionally, such classification of up to 90% of the activities of daily living of manual wheelchair users with SCI was found to be clinically useful [24–26]. Prior investigation of classifying instability of manual wheelchairs combined use of IMUs and machine learning to differentiate destabilizing conditions [27]. In contrast, our research utilizes a simple threshold-based method to detect and classify destabilizing events based on characteristics of the inertial signature of the wheelchair to automatically trigger activation of the appropriate muscles and determine its impact on seated posture.

The static and dynamic tipping stability of the wheelchair-user system has been examined in modeling studies [28–31]. Li et al. [30] derived an equation to calculate the critical velocity at which unrecoverable forward tipping will occur due to a collision. The equation and parameters used for this paper are explained in the methods section below. Similarly, Bruno’s collision simulations [28] released a powered wheelchair from the top of a ramp to passively ride down into a barrier at the end, and found that the system is expected to fully tip forward when entering a collision at 1.74 m/s. Cooper et al. [31] developed the following equation to predict rollover during a destabilizing turn, based on the initial velocity and radius of the turn. This equation is also explained in the methods section below. Such wheelchair modeling studies inform the design of wheelchairs for safe operation and define their inherent mechanical limits of stability, but do not react to potentially destabilizing events or assist wheelchair users in maintaining and/or regaining stable seated postures when they occur.

The purpose of this study was to determine the feasibility and preliminary performance of a threshold-based system that utilizes inertial measurements to detect destabilizing collisions and sharp turns in manual wheelchair users with SCI and provides an appropriate stimulated response of the hip and trunk muscles through implanted pulse generators (IPGs) or surface stimulation to enhance recovery of a stable seated posture, thereby potentially decreasing the risk of injurious loss of balance and falls. IPGs or surface stimulators electrically excite the motor nerves with adjustable pulse amplitudes and durations to activate the otherwise paralyzed hip and trunk muscles and generate the desired movements. It has been shown that FNS requires roughly 100 ms to generate torque in the muscles [32]. Therefore, this system must act in real time to detect or predict the onset of such events to allow for earliest possible application of stimulation before instability reaches an irreversible state. We hypothesize that applying FNS at the onset of a destabilizing event will increase trunk stability, maintain erect posture, and improve users’ perceptions of safety during daily activities in their wheelchairs. This study analyzed detection accuracy, detection delay, and restoration of trunk stability with and without neural stimulation delivered by a threshold-based system during destabilizing collisions and turns in manual wheelchair users with SCI.

## Methods

### Preliminary simulations and crash dummy testing

To design an appropriate and safe experimental set-up for testing the impact of destabilizing crashes and sharp turns, mathematical models of the wheelchair-user system were utilized to simulate collision and turning events [29, 30]. The equation derived by Li et al. [30] to calculate the critical velocity at which unrecoverable forward tipping will occur due to a collision follows:

1 $$ {v}_{crit}=\sqrt{\frac{2g{\left|{d}_{cm}\right|}^2}{M{h}^2}\left({I}_{cm}+M{\left|{d}_{cm}\right|}^2\right)\left(1-\mathit{\cos}\left({\theta}_{crit}-\theta \right)\right)\ } $$

In this equation, *d*_*cm*_ is the displacement vector from the front caster to the center of gravity, *M* is the combined mass of the wheelchair-user system, *h* is the height of the center of gravity above the ground, *I*_*cm*_ is the system moment of inertia, *θ*_*crit*_ is the critical angle at which tipping the wheelchair to this degree will be recovered at in static conditions, and *θ* is the angle of the slope on which the wheelchair is placed. Table 1 outlines the parameters used to calculate the critical velocity from Eq. 1. These parameters were measured from the same wheelchair that was used by all the participants during laboratory testing and a user mass of 75 kg was assumed. From this calculation, the critical velocity for tipping of the wheelchair-user system was expected to occur at 1.6 m/s. To avoid tipping and ensure subject safety, a slightly lower velocity of 1.5 m/s was chosen for experimental sessions. This choice of velocity was confirmed in a computer simulation of the collision event in Simwise 4D (Design Simulation Technologies, Canton, MI) in which a model of a 75-kg person riding in a 15-kg wheelchair descended a ramp with a 5^o^ incline. The wheelchair operator was represented by a simple multi-joint model consisting of revolute knee and hip joints, with nominal mass parameters of the thighs and lumped head, arms and trunk. Terminal speeds were varied by adjusting the starting position on the simulated ramp. Simulation results confirmed that an impact at 1.5 m/s generated peak deceleration on the order of 4 g, which was sufficient to passively lift the thighs from the seating surface and flex the trunk forward. Cooper et al. [31] developed the following equation to predict rollover during a destabilizing turn, based on the initial velocity and radius of the turn:

2 $$ {v}_{crit}=\sqrt{\frac{rgD}{x}} $$

**Table 1:** Parameters for Critical Velocity Calculations

Collision (Eq. 1)		Turns (Eq. 2)	
d_cm_	0.81 m	r	0.635 m
M	90 kg	D	0.30 m
h	0.71 m	x	0.71 m
I_cm_	9.5 kg-m^2^		
θ_crit_	31°		
θ	5°		

In this equation, *r* is the radius of the turn, *D* is half the width of the wheelchair, and *x* is the height of the center of gravity above the seat of the chair. Using the parameters outlined in Table 1 for Eq. 2, a system entering a turn of 25-in. (63.5 cm) radius at 1.5 m/s is expected to be destabilizing without causing the chair to roll over.

**Fig. 1:**
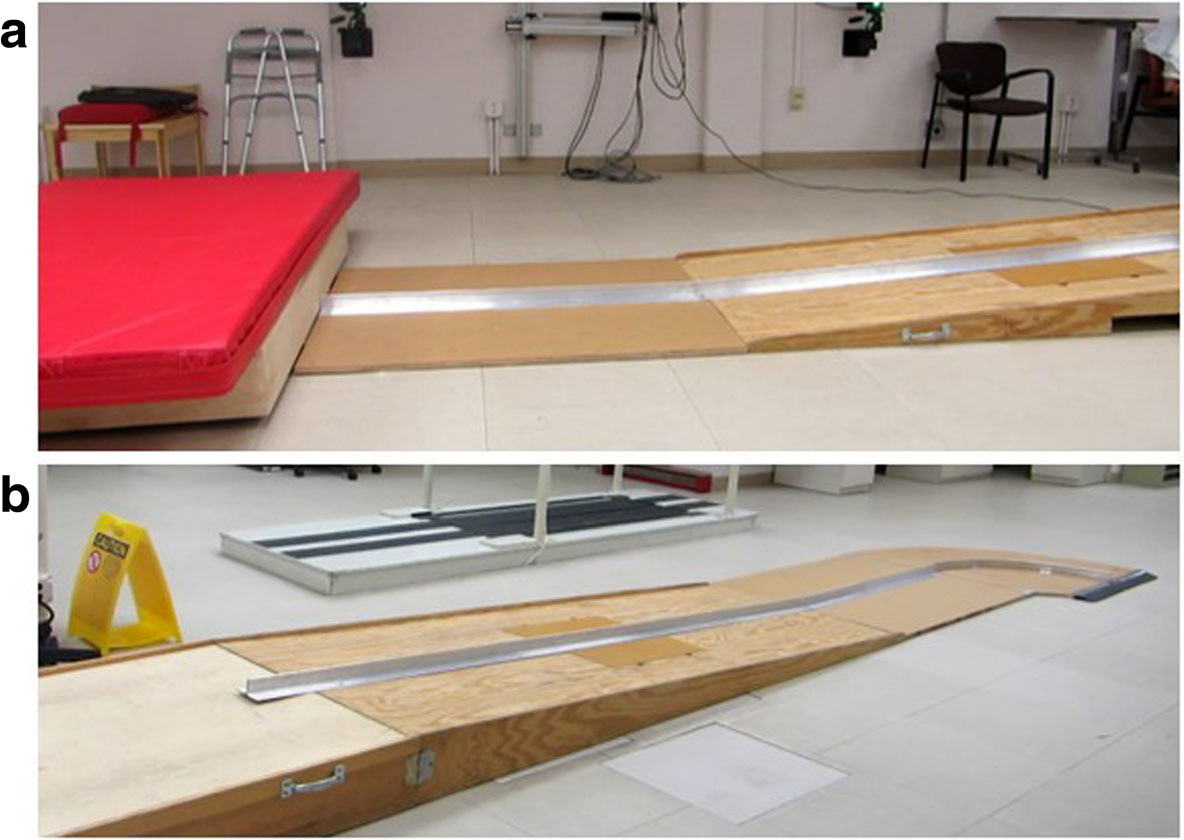
Experimental Setup. Experimental set-up of the ramp with a guidance ramp for (**a**) collisions and (**b**) 90-degree turns

A 2-m ramp with 5^o^ incline was constructed for the experimental set up. A guidance track was mounted on top of the ramp, which ended with a 90 degree turn of 25-in. (63.5-cm) radius. Roller bearings were installed beneath the frame of a standard wheelchair (GP Series, Sunrise Medical LLC, Fresno, CA) to guide descent and turning and achieve a consistent velocity at impact or time of turn. A bumper was also attached to the footplate of the wheelchair to protect the feet during all experiments. A barrier was erected 2 m after the turn to suddenly stop the wheelchair and rider and thus simulating a collision. The experimental set-up is shown in Fig. 1.

**Fig. 2:**
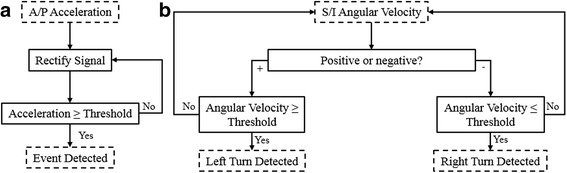
Detection Algorithms. **a** Collision detection algorithm **b** Turn detection algorithm

Collisions and rapid turns were performed with a 6′1″, 165-lb (1.85 m, 74.8-kg) Rescue Randy crash dummy (Dummies Unlimited Inc., Pomona, CA) seated in the manual wheelchair to develop the event detection algorithms and verify safety prior to human testing. The three-dimensional position coordinates of the wheelchair and crash dummy were tracked using a 16-camera motion capture system (VICON MX, Oxford Metrics, Oxford, UK), sampling at 100 Hz. Ten reflective markers were placed on the wheelchair-user system: two markers on the rear cross bar of the chair, one at each wheel center, one above each caster, one on each shoulder, and one on the sternum and C7 vertebra. A wireless IMU, containing a CMA3000-D01 accelerometer from Texas Instruments (Dallas, TX) and a LSM330DLC gyroscope from ST Microelectronics (Geneva, Switzerland), was placed on the center of the rear cross bar of the wheelchair. The IMU sampled the tri-axial acceleration and angular velocity of the wheelchair at 100 Hz. For each trial, the wheelchair with dummy rider was released from the top of the ramp, passively rode down the guidance track into a 90 degree turn at the lowest part of the incline and ended with a sudden stop to stimulate a collision. Twenty trials of collisions, right turns, and left turns were collected each. Mean peak AP acceleration and superior-inferior (SI) angular velocity were found to repeatedly and reliably represent collisions and turns, respectively, and were therefore utilized in subsequent experiments to detect the events. Simple threshold-based event detection algorithms illustrated in Fig. 2 were devised based on these preliminary tests. Calculation of thresholds for the algorithms used for participants with SCI is further explained in the following section.

### SCI testing

A total of four participants (S1-S4) with low thoracic or high cervical spinal cord injuries participated in this study. To be eligible for participation, subjects must have been 21 years of age or older, sustained a mid-cervical or thoracic spinal cord injury (C4-T12), classified as A through C on the ASIA Impairment Scale, scored less than 4 on the Ashworth Scale for Spasticity and the Modified Ashworth Scale for hypertonia, and able to read and speak English. All subjects signed consent forms approved by the local institutional review board before any experiments were conducted. Table 2 summarizes the clinical characteristics of each participant. Each had received a surgically implanted neuromuscular stimulator to activate the knee, hip and trunk extensor muscles with implanted intramuscular or nerve cuff electrodes as part of participation in other projects in our laboratory [33–38]. Details of the implanted motor system neuroprostheses are described elsewhere [39–43], and consisted of 8, 12, or 16 channel IPGs that delivered asymmetrical charge-balanced current controlled stimulus waveforms with pulse amplitudes (0 to 20 mA) selectable for each channel and variable pulse durations (0 to 250 μsec) and frequencies (0 to 20 Hz) set on a pulse-by-pulse basis. Intramuscular electrodes [44] were inserted at the T12-L2 spinal nerves to activate the paraspinal muscles, and at the motor points of the gluteal and hamstring muscles. Spiral nerve cuff electrodes [45] were also installed on the femoral nerves were in all subjects to activate the quadriceps. In the ensuing experiments, subject S3 utilized surface stimulation rather than his implanted system because of technical difficulties that could not be resolved in time to complete the testing and to begin to examine the generalizability of the approach for the larger SCI population without implanted electrodes and pulse generators.

**Table 2:** Clinical summary of study participants

Participant	Age	Gender	Injury Level	AIS Grad	Date of Injury	Date of Implant
S1	51	M	T3	A	10/11/2002	2/24/2015
S2	41	F	T4	A	2/13/2012	11/20/2014
S3	59	M	T4	B	3/9/2008	1/16/2012
S4	44	F	C7	B	3/13/1998	8/26/2010

For each event, participants first went through 20 calibration trials to determine threshold values of AP acceleration and SI angular velocity indicative of collisions and turns. The same wheelchair was used by all subjects. Participants also wore helmets and loosely fitting seat belts to ensure safety. For collisions, a subject’s threshold was calculated as the mean peak of the absolute value of AP acceleration minus two standard deviations from his/her own 20 calibration trials. Turn thresholds were calculated in the same manner as the mean peak of the absolute value of the SI angular velocity minus two standard deviations. Right and left turn thresholds were equal in magnitude, but opposite in sign. A positive threshold detected a left turn, whereas a negative threshold classified a right turn.

**Fig. 3:**
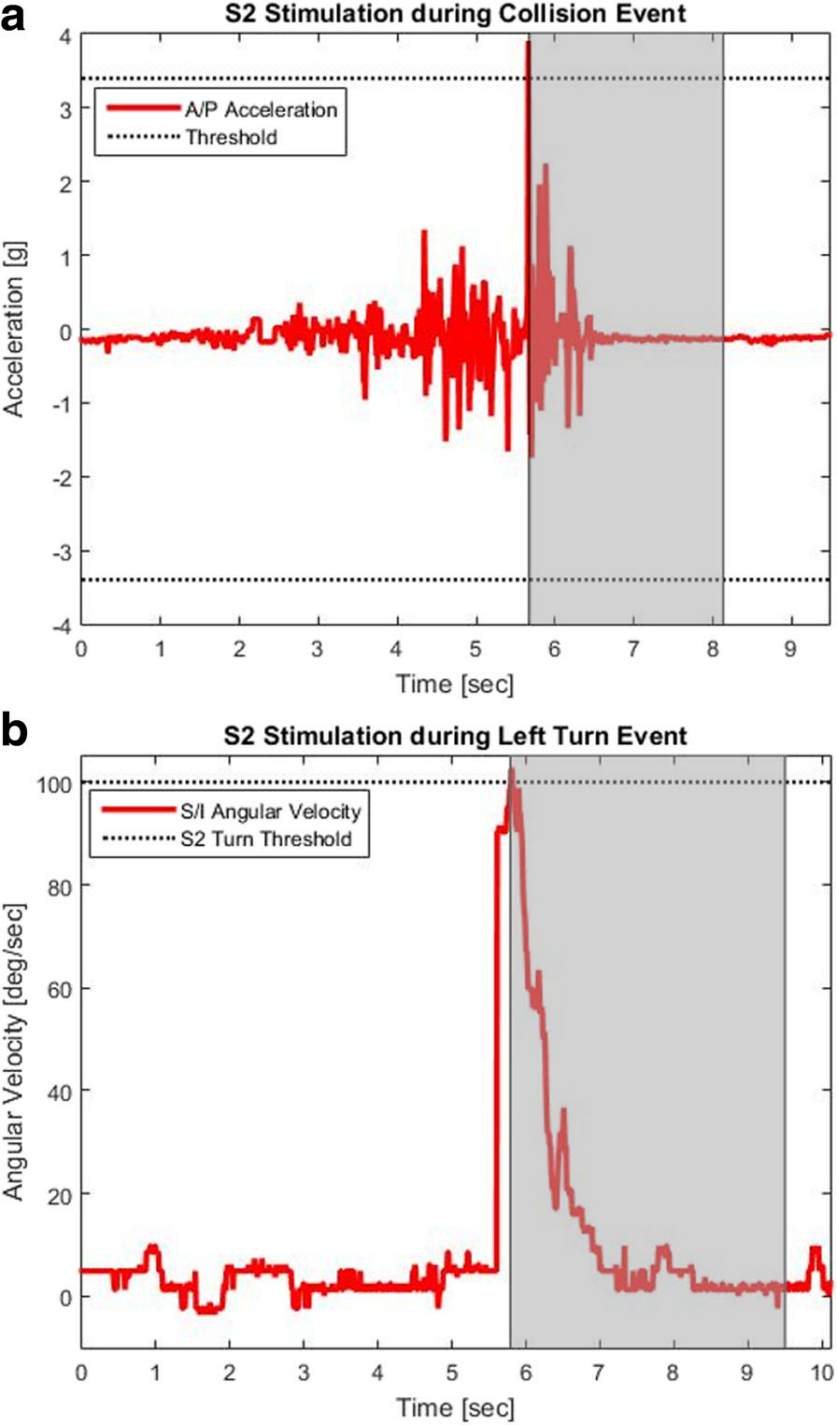
Stim Timings for S2. **a** AP acceleration of a collision event for S2, with stimulation activated during the shaded portion (**b**) SI angular velocity of a left turn event for S2, with stimulation activated during the shaded portion. Right turns are symmetrical to left turns over the x-axis

The real-time decision algorithms for collisions and turns, shown in Fig. 2, were created in Simulink (The Mathworks Inc., Natick, MA), which received the IMU signals from the wheelchair, compared them to the subject-specific thresholds, and delivered stimulation to the muscle groups at the levels customized for each subject when the threshold was exceeded. The participants manually turn the stimulation off once they restored a stable, erect posture. The relative timings of stimulation to inertial measurements are depicted in Fig. 3. For forward collisions, maximal stimulation levels were generally delivered to the trunk (lumbar erector spinae) and hip (gluteus maximus, posterior adductor, and/or hamstrings) extensor muscles to resist forward flexion and assist return to upright sitting. For turns, the quadratus lumborum on the inside of the turn, and the hip extensor on the outside of the turn were activated to resist lateral displacement of trunk and pelvis. Table 3 summarizes the algorithm thresholds and muscles stimulated for each subject during each event.

**Table 3:** Algorithm thresholds and muscles stimulated during collision and turn events for each participant

	Collisions			Turns			
	Mean of the Peak AP Acceleration	Algorithm Threshold	Muscles Stimulated	Mean of the Peak SI Angular Velocity	Algorithm Threshold	Right Turn: Muscles Stimulated	Left Turn: Muscles Stimulated
S1	3.93 ± 0.08 g	3.76 g	PA, GM, ES, QL	126.23 ± 13.8 deg./s	98.63 deg./s	Right ES, Left GM, Right QL, Right/Left PA	Left ES, Right GM, Left QL, Right PA
S2	3.83 ± 0.22 g	3.39 g	PA, GM, ES, QL, IP	105.5 ± 2.73 deg./s	100.04 deg./s	Right ES, Right/Left GM, Right QL, Right/Left PA, Right/Left IP	Left ES, Right/Left GM, Right/Left PA, Right/Left IP
S3	3.87 ± 0.19 g	3.48 g	GM, ES, QL, HS	109.77 ± 4.88 deg./s	100.00 deg./ s	Left GM, Left HS	Left ES, Right GM, Right HS
S4	–	–	–	105.30 ± 4.08 deg./s	97.10 deg./s	Right ES, Right/Left GM, Right, QL, Right/left PA	Left ES, Right/Left GM, Left QL, Right/Left PA

Labels: *ES* Erector Spinae, *GM* Gluteus Maximus, *QL* Quadratus Lumborum, *PA* Posterior Adductor, *IP* Iliopsoas, *TF* Tensor Fascia Latae, *HS* Hamstring

Once unique thresholds and patterns of stimulation were determined, participants went through five trials with event-based stimulation and five trials without the controller active. The accuracy of classification and detection delay, defined as the difference between event onset determined by the VICON motion capture system and detection of the event by the algorithm, were computed for each trial to define the efficacy of the classifier. Maximum trunk angle in the AP direction for collisions, medial-lateral (ML) direction for turns, and return time to erect posture were calculated to understand how event-triggered stimulation affected trunk stability. The statistical significance of changes to the trunk angle and return time was determined using repeated measures ANOVA test in Matlab (The Mathworks Inc., Natick, MA) with *p* < 0.05 denoting significance. The Usability Rating Scale (URS), a seven-point ordinal scale ranging from “very difficult” (− 3) to “very easy” (3), was applied to quantify users’ feeling of safety and stability after each trial [46]. For the purposes of this study, the wording of the URS was adjusted to rate events as “very unstable” (− 3) to “very stable” (3). The non-parametric Wilcoxon Signed Rank Test (Laerd Statistics) was used for statistical analysis of the URS data with p < 0.05 denoting significance. Collision data was only collected and analyzed for Subjects S1, S2, and S3. For turns, all four participants underwent testing with real-time event detection to determine classification accuracy.

This feasibility study took the form of a series of case studies with participants acting as their own concurrent controls. Primary comparisons were made between baseline (no stimulation) and experimental condition (with event detection and stimulation) for each individual subject without pooling data across participants for group comparisons. Q-Q plots confirmed normalcy of the repeated measures of the outcome variables for each subject, which were treated as independent observations as per standard single-subject research techniques [47–49]. The order of testing was randomized, subjects were blinded as to the condition (baseline or triggered stimulation) and long periods of rest were interspersed between successive trials.

## Results

### Crash dummy testing

At the instant of collision, the acceleration spiked, reaching ±4 g, similar to the peak shown in Fig. 3a. The magnitude of this peak provided a unique feature observed in this event only. Similarly, in data collected during right and left turns, the angular velocity in the SI direction reached magnitudes over 100 deg./s, which provided a distinct feature that was unique for this event, similar to that of Fig. 3b. Left and right turns could be distinguished by the sign of the peak: left turns exhibited a positive peak, while right turns exhibited a negative peak.

### SCI testing

The efficacy of the classifier for each subject in terms of detection accuracy and delay for collisions, right turns, and left turns are outlined in Table 4. Across the three subjects who participated in the collision experiments, the classifier accurately detected 93% of the trials. For S3, one trial was a false negative, in which the classifier did not recognize a collision as the maximum AP acceleration did not exceed the detection threshold of 3.48 g. The false negative is likely due to a faulty accelerometer signal. Across all three subjects, the average detection delay was 88 ms ± 48 ms. During right turn trials, the classifier accurately classified 93% of the trials. For S2, one trial was a false negative, in which the classifier did not recognize a right turn as the SI angular velocity did not exceed the detection threshold of 100 deg./s. For left turns, the classifier accurately detected 100% of the trials. The average detection delay across all four subjects during right and left turns was 342 ± 73 ms.

**Table 4:** Event detection system

Participant	Collision		Right Turn		Left Turn	
	Detection Accuracy	Detection Delay (ms)	Detection Accuracy	Detection Delay (ms)	Detection Accuracy	Detection Delay (ms)
S1	5/5	110 ± 80	5/5	146 ± 45	5/5	190 ± 100
S2	5/5	40 ± 20	4/5	365 ± 43	5/5	420 ± 30
S3	4/5	118 ± 14	5/5	260 ± 10	5/5	370 ± 50
S4	–	–	5/5	320 ± 20	5/5	343 ± 88

**Fig. 4:**
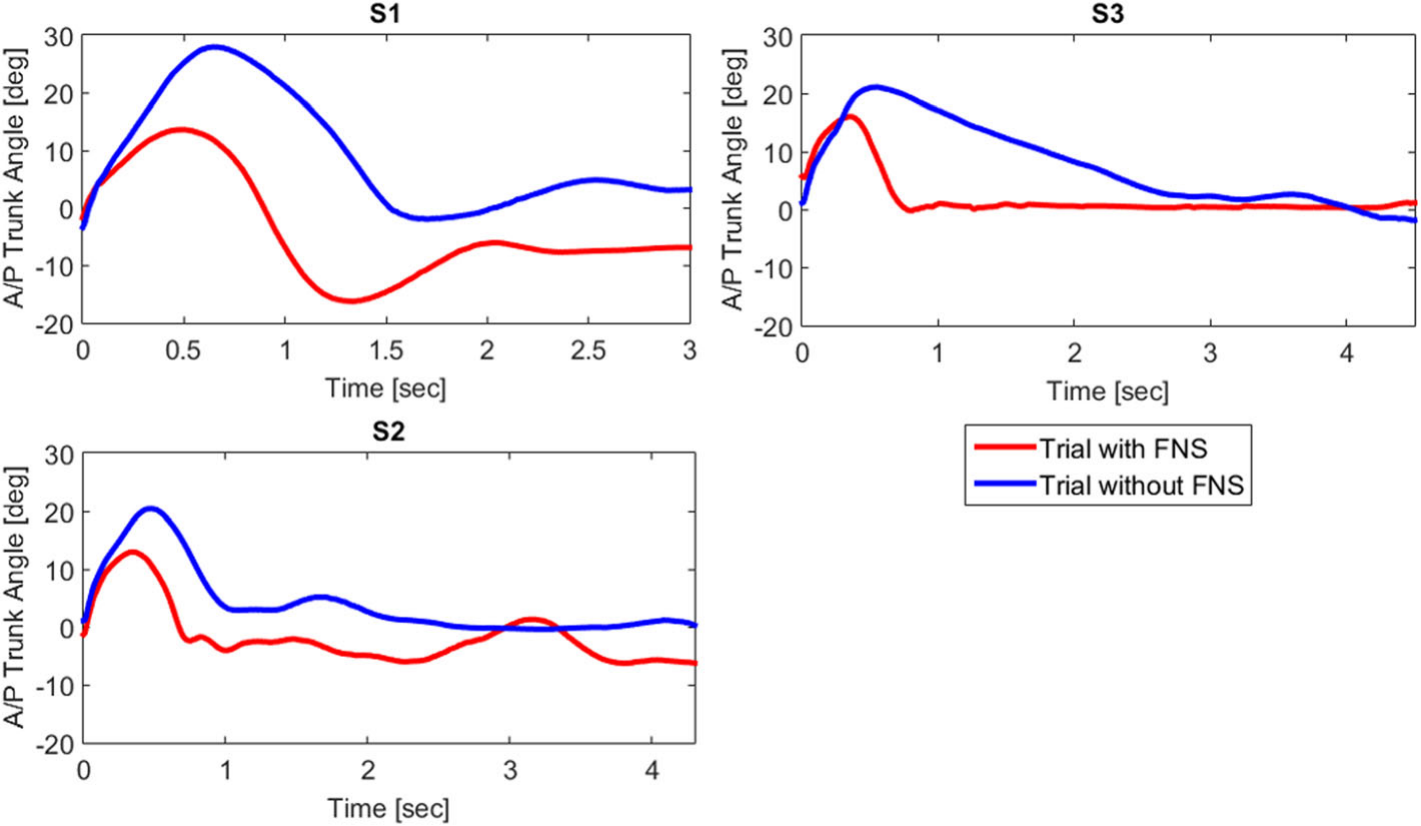
SCI AP Trunk Angles during Collision. The AP trunk angle during a collision trial with FNS (red lines) and without FNS (blue lines) for S1, S2, and S3. Zero seconds marks the start of the collision event

**Fig. 5:**
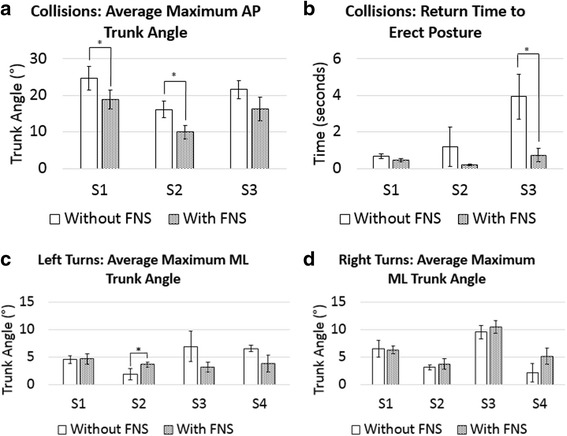
Comparison of Maximum Trunk Angles and Return Time to Erect Posture with and without FNS*.* **a** Average maximum AP trunk angles during collision trials with FNS versus without FNS (**b**) Average return time to an erect seated posture during collision trial with and without FNS (**c**) Average maximum ML trunk angles during right turns with FNS and without FNS (**d**) Average maximum ML trunk angles during left turns with FNS and without FNS (* denotes statistical significance with *p* < 0.05)

**Fig. 6:**
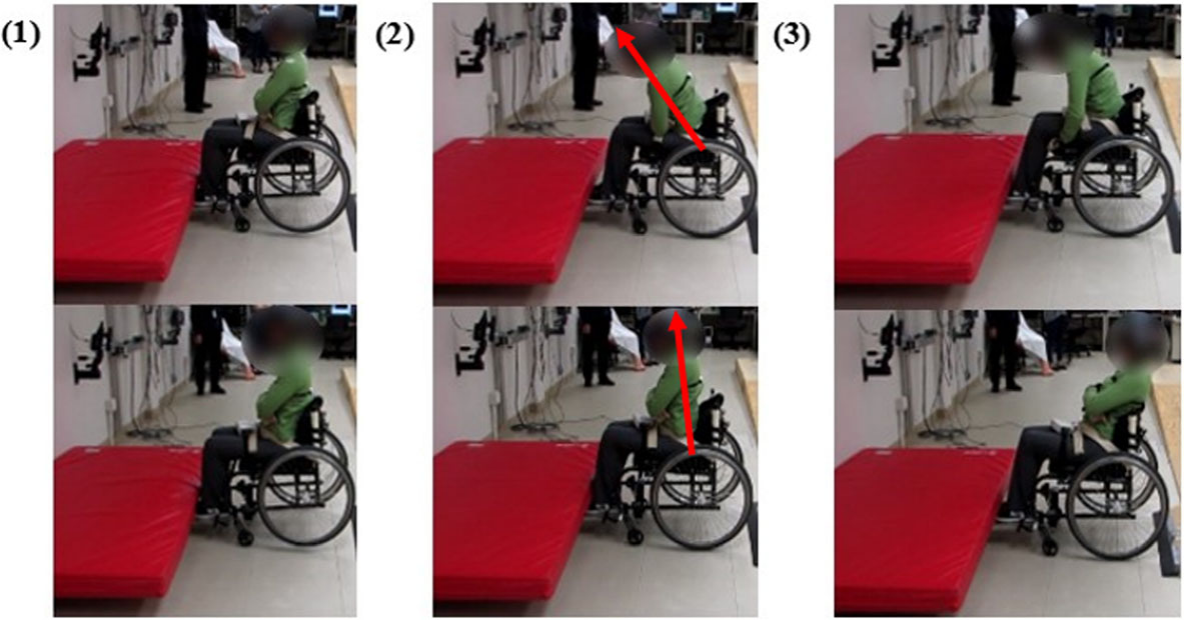
Collision Event. Collision trial without stimulation (top row) and with stimulation (bottom row). The images include: (1) Initial impact: wheelchair contacts obstacle and rear wheels lift off the ground (2) Maximum AP trunk angle (3) Return to erect posture, which requires manual re-adjustment in the trial without stimulation

The effects of FNS on trunk stability for S1, S2, and S3 in terms of maximum AP trunk angle, return time, and URS median are summarized for collisions in Table 5. Figure 4 shows a decrease in the maximum AP trunk angle across the subjects when stimulation was applied. The average maximum AP trunk angle decreased for S1 from 24.78° ± 2.62° without FNS to 18.88° ± 3.23° with FNS and for S2 from 16.10° ± 1.76° without FNS to 9.92° ± 2.24° with FNS, which were both statistically significant differences (*p* < 0.05). Figure 5a and b display the differences in maximum AP trunk angles and return time to erect posture, respectively, with and without FNS for each subject. Figure 6 shows collision trials with and without stimulation for a participant. In general, the URS scores for S1 and S2 for trials with FNS were higher than trials without FNS. However, none of these results showed a significant difference based on the Wilcoxon Signed Rank Test.

**Table 5:** Collision results

	S1			S2			S3		
	With FNS	Without FNS	*P*-value	With FNS	Without FNS	*P*-value	With FNS	Without FNS	*P*-value
Average Max AP Trunk Angle (°)	18.88 ± 3.23	24.78 ± 2.62	0.03*	9.92 ± 2.24	16.1 ± 1.76	0.005*	16.23 ± 2.52	21.60 ± 3.21	0.08
Average Return Time to Erect (s)	0.45 ± 0.07	0.65 ± 0.13	0.05	0.20 ± 0.05	1.18 ± 1.07	0.18	0.73 ± 0.37	3.94 ± 1.23	0.007*
URS Median	1	0	0.1	3	2	0.1	1	1	1

*denotes statistical significance with *p* < 0.05

**Fig. 7:**
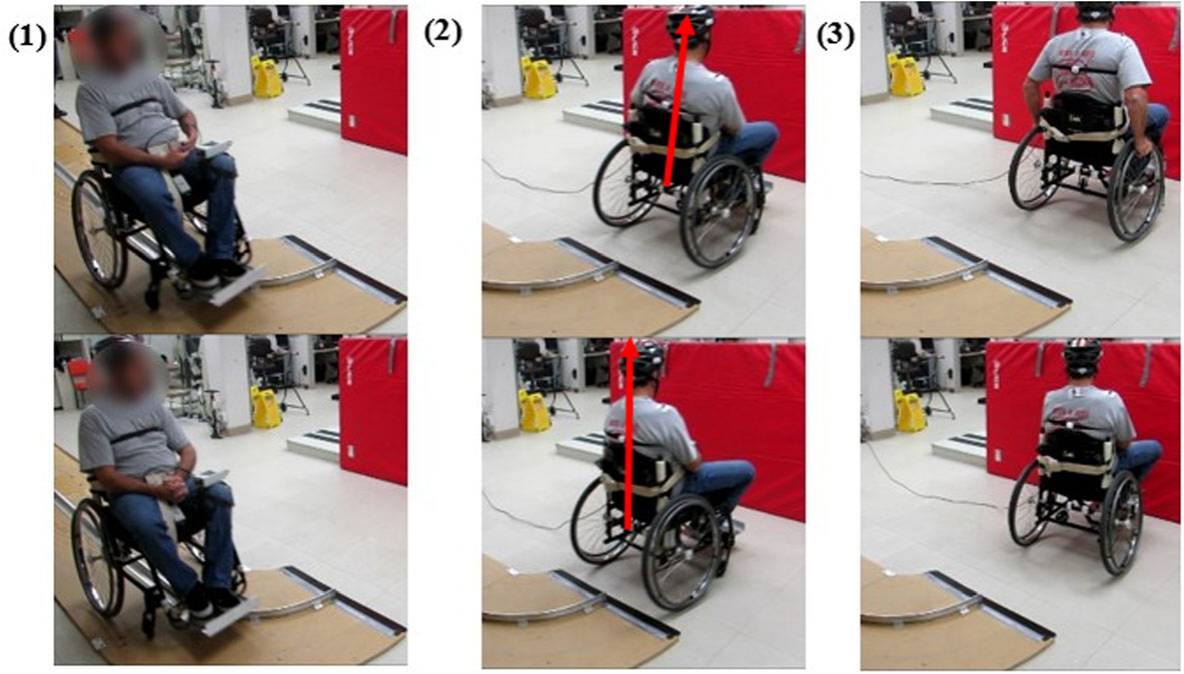
Turn Event. Left turn trial without stimulation (top row) and with stimulation (bottom row). The images include: (1) Initial turn: wheelchair enters turn and detection is triggered (2) Maximum ML trunk angle: without stim, the trunk leans out of the turn due to inertia, whereas with stim, the trunk leans into the turn (3) Return to erect posture, which requires manual re-adjustment in the trial without stimulation

Table 6 summarizes the effects of FNS on trunk stability during right and left turns for all four subjects. On average, S2 experienced increased maximum ML trunk angles with stimulation during right and left turns, as did S3 during right turns. This increase was significant for S2 during left turns, with an average trunk angle of 3.63° ± 0.46° with FNS and 1.84° ± 1.01° without FNS (*p* = 0.02). Figure 5c and d display the differences in maximum ML trunk angles with and without FNS for each subject. Figure 7 shows left turns with and without stimulation for a participant. The URS scores for S4 were significantly increased with FNS during right turns (Z = − 2.121, *p* = 0.03). The participants said the FNS triggered too late within the turn for the stimulation to be effective, which resulted in the URS scores for trials with FNS and without FNS to be similar.

**Table 6:** Right and left turn results

		S1			S2			S3			S4		
		With FNS	Without FNS	*P*-value	With FNS	Without FNS	*P*-value	With FNS	Without FNS	*P*-value	With FNS	Without FNS	*P*-value
Right Turns	Max ML Trunk Angle (°)	6.24 ± 0.71	6.48 ± 1.59	0.81	3.72 ± 0.97	3.11 ± 0.45	0.36	10.46 ± 1.18	9.55 ± 1.20	0.4	2.15 ± 1.65	5.11 ± 1.49	0.06
	URS Median	-1	0	0.1	−3	−3	1	2	2	1	1	−1	0.03*
Left Turns	Max ML Trunk Angle (°)	4.66 ± 0.93	4.52 ± 0.74	0.84	3.63 ± 0.46	1.84 ± 1.01	0.02*	3.15 ± 0.86	6.91 ± 2.77	0.09	3.80 ± 1.51	6.51 ± 0.58	0.06
	URS Median	0	0	0.5	0	0	0.08	2	2	1	0	−1	0.06

*denotes statistical significance with *p* < 0.05

## Discussion

A threshold-based system for maintaining trunk stability for manual wheelchair users was designed and tested in four subjects with SCI. The system designed and implemented in this study classified destabilizing collisions with an accuracy greater than 90%, which aligns with previous clinical applications of event detection devices [24]. For S1 and S2, the stimulation significantly decreased the maximum AP trunk angle upon the collisions. Because of technical difficulties generating stimulation at the back extensor muscles in S3, FNS during these trials was limited to hip extensors. This limitation may have negatively affected the efficacy of the system to restore trunk stability for this subject. Despite attempts to control the speed at impact with the ramp and barrier set-up, the subject did not always achieve the required velocity of 1.5 m/s prior to collision to produce the desired deceleration profile which would have exceeded threshold and successfully triggered stimulation. A lower threshold or more precise control of the velocity at impact may have improved classification performance above the 93% accuracy observed.

During turns, the classifier also achieved detection accuracy greater than 90%. On average, delay in detection of right and left was 342 ± 73 ms, causing significant delay for stimulation to be activated. With an additional time delay of 100 ms for FNS to generate maximum torque [32], the recruited muscles would not be activated fast enough to be provide the desired response in this application. The participants noticed this delay, stating their trunks felt more stable once stimulation was applied, but the stimulation became effective too late into the turn. One false negative was experienced for S3 during right turns. The thresholds for turns could be less conservative, allowing higher detection accuracy and possibly decreasing the delay of detection. The maximum ML trunk angle was not significantly decreased for any of the participants, and in some cases, increased when stimulation was applied. This may be a result of participants preparing for the sharp turns by using head and shoulder movements to lean into the turn and prevent falling out during trials with and without stimulation applied. To overcome the delay, future studies should consider monitoring the angular acceleration, rather than the angular velocity. Upon post-processing of the data across all four subjects, turn detection using angular acceleration achieved detection within 17.17 ± 11.40 ms. Alternatively, it may be necessary to activate stimulation before the event to effectively restore trunk stability. Investigation in other sensor modalities, such as ladar [50–53] and vision-based methods [53, 54], or sonar readings [55] used to classify terrain in autonomous vehicle and robotic navigation applications, would be necessary to predict destabilizing events before they occur.

During a false negative classification, the controller does not apply FNS during a destabilizing event. In this case, if no secondary methods to maintain stability are in place, the user can fall out of the wheelchair and sustain injuries. Alternatively, a false positive classification would activate FNS during a period of stability. This activation of FNS may surprise the user, but will not produce any injurious consequences. Thus, in the event of a misclassification of this event detection controller, we would prefer false positive classifications.

It should be noted that because these trials were conducted in a laboratory setting and the events were controlled, some subjects assumed safety regardless of whether FNS came on or not as they understood the experiment would be designed to avoid potential injury. This assumption was reflected in participants’ URS scores, particularly S3, who rated all trials with and without FNS as moderately or very stable. Also, in this setting, we only tested the collision and turning events in the ways we designed them, which may not reflect how they occur in real life situations. For example, if an oblique collision happened, deceleration of this event would be detected in both the ML and AP directions, and may not be recognized as a collision using the algorithm discussed in this paper. The accelerometers utilized in this experiment were limited to a range of ±4 g. With this restriction, the mean and standard deviations used to define thresholds may be saturated as the AP acceleration often reached these limits during collision testing, though it is expected for 4 g to sufficiently define a destabilizing collision.

Previous work by Audu et al. [17] has shown success using feedback controlled neuroprostheses for restoring an erect posture in people with SCI in response to external perturbations. Additionally, Patel et al. [18] showed that a powered-wheelchair neuroprosthesis which activated FNS of trunk muscles of able-bodied participants in response to perturbations in the AP direction decreased trunk displacement and velocity. The work presented in this paper shows promise for an event detection system that could decrease wheelchair-related injuries, while increasing users’ independence without the need for restrictive straps or seatbelts. To our knowledge, this is the first study that applies neural stimulation in response to potentially destabilizing wheelchair events detected by wheelchair-mounted IMUs in individuals with SCI during simulated crashes and sharp turns. This system was tested with both implanted and surface stimulation, suggesting its potential applicability for a wider population beyond implant recipients. Larger scale studies with significantly mores subjects are necessary to determine the generalizability of the results and transcend the single-subject feasibility research design.

Future tests will be conducted utilizing accelerometers with higher ranges to fully understand the nature of the signals during collisions. This controller will also be tested outside of the laboratory setting for use in the community and during activities of daily living to understand the clinical significance of this device. Additionally, future tests will examine both a turn and collision simultaneously to ensure the controller responds appropriately to restore trunk stability during the combination of events. Lastly, algorithms will be developed for other potentially destabilizing events, such as rough terrain, bumps, and ramps. With increased trunk stability, manual wheelchair users may feel more confident traversing through unfamiliar environments and care should be exercised to capture such subjective perceptions of safety in addition to biomechanical measures.

## Conclusion

A new threshold-based system that accurately detected potentially destabilizing events and triggered activation of the paraspinal and hip muscles to improve stability and compensate for the disturbances was designed and tested with manual wheelchair users paralyzed by spinal cord injuries. This controller, which monitored the AP acceleration of the wheelchair to detect collisions and SI angular velocity to detect turns, achieved accuracy greater than 90%. Two of the three subjects who received stimulation to the hip and trunk extensor muscles during the collision events experienced increased trunk stability manifested by decreased forward lean and more rapid restoration of erect posture from a flexed position. Larger delays in detection for turns based on angular velocity negatively affected the ability of the controller to return the trunk to erect from a lateral bend, but further investigation with less conservative thresholds and other sensor modalities may fix this issue. The success of the event detection and the ability to apply stimulation to restore trunk posture with neural stimulation in response to destabilizing events shows promise for a device which increases safety and stability of manual wheelchair users during everyday activities.

## Abbreviations

ANOVA: Analysis of variance
AP: Anterior-posterior
ES: Erector spinae
FNS: Functional neuromuscular stimulation
GM: Gluteus maximus
HS: Hamstring
IMU: Inertial measurement unit
IP: Iliopsoas
IPG: Implanted pulse generator
ML: Medial-lateral
ms: Millisecond
PA: Posterior adductor
QL: Quadratus lumborum
SCI: Spinal cord injury
SI: Superior-inferior
TF: Tensor fascia latae
URS: Usability rating scale

## References

[1] Spinal Cord Injury (SCI) Facts and Figures at a Glance. In: The National Spinal Cord Injury Statistical Center. The University of Alabama at Birmingham Department of Physical Medicine and Rehabilitation. https://www.nscisc.uab.edu/Public/Facts%20and%20Figures%20-%202017.pdf. Accessed 7 June 2017.

[2] Complete Public Version of the 2013 Annual Statistical Report for the Spinal Cord Injury Model System. In: The National Spinal Cord Injury Statistical Center. The University of Alabama at Birmingham Department of Physical Medicine and Rehabilitation. https://www.nscisc.uab.edu/PublicDocuments/reports/pdf/2013%20NSCISC%20Annual%20Statistical%20Report%20Complete%20Public%20Version.pdf. Accessed 7 June 2013.

[3] Anderson KD (2004). Targeting Recovery: Priorities of the Spinal Cord-Injured Population. J Neurotrauma.

[4] Milosevic M, Masani K, Kuipers MJ, Rahouni H, Verrier MC, McConville KM, Popovic MR (2015). Trunk Control Impairment is Responsible for Postural Instability during Quiet Sitting in Individuals with Cervical Spinal Cord Injury. Clin Biomech (Bristol, Avon).

[5] Xiang H, Chany AM, Smith GA (2006). Wheelchair Related Injuries: Treated in the US Emergency Departments. Inj Prev.

[6] Gavin-Dreschnack D, Nelson A, Fitzgerald S, Harrow J, Sanchez-Anguiano A, Ahmed S, Powell-Cope G (2005). Wheelchair-related Falls: Current Evidence and Directions for Improved Quality Care. J Nurs Care Qual.

[7] Gaal RP, Rebholtz N, Hotchkiss RD, Pfaelzer PF (1997). Wheelchair rider injuries: causes and consequences for wheelchair design and selection. J Rehabil Res Dev.

[8] Medola FO, Elui VMC, Santana CS, Fortulan CA (2014). Aspects of Manual Wheelchair Configuration Affecting Mobility: A Review. J Phys Ther Sci.

[9] Kirby RL, Sampson MT, Thoren FAV, Macleod DA (1995). Wheelchair Stability Effect of Body Position. J Rehabil Res Dev.

[10] Kirby RL, Ackroyd-Stolarz SA (1995). Wheelchair Safety-Adverse Reports to the United States Food and Drug Administration. Am J Phys Med Rehabil.

[11] Chaves ES, Cooper RA, Collins DM, Karmarkar A, Cooper R (2007). Review of the Use of Physical Restraints and Lap Belts with Wheelchair Users. Assist Technol.

[12] Kamper D, Barin K, Parnianpour M, Weed H (1999). Preliminary Investigation of Lateral Postural Stability of Spinal Cord-Injured Individuals Subjected to Dynamic Perturbations. Spinal Cord.

[13] Minkel JL (2000). Seating and Mobility Considerations for People with Spinal Cord Injury. Phys Ther.

[14] Triolo RJ, Nogan-Bailey S, Miller ME, Lombardo LM, Audu ML (2013). Effects of Stimulating Hip and Trunk Muscles on Seated Stability, Posture, and Reach after Spinal Cord Injury. Arch Phys Med Rehabil.

[15] Vette AH, Wu N, Masani K, Popovic MR (2015). Low-Intensity Functional Electrical Stimulation Can Increase Multidirectional Trunk Stiffness in Able-Bodied Individuals during Sitting. Med Eng Phys.

[16] Milosevic M, Masani K, Wu N, McConville KM, Popovic MR (2015). Trunk Muscle Co-activation using Functional Electrical Stimulation Modifies Center of Pressure Fluctuations during Quiet Sitting by Increasing Trunk Stiffness. J Neuroeng Rehabil..

[17] Audu ML, Lombardo LM, Schnellenberger JR, Foglyano KM, Miller ME, Triolo RJ (2015). A Neuroprosthesis for Control of Seated Balance after Spinal Cord Injury. J Neuroeng Rehabil.

[18] Patel K, Milosevic M, Nakazawa K, Popovic MR. Wheelchair Neuroprosthesis for Improving Dynamic Trunk Stability. IEEE Trans Neural Syst Rehabil Eng. 2017. doi:10.1109/TNSRE.2017.2727072. 10.1109/TNSRE.2017.2727072 28715333

[19] Masani K, Sin VW, Vette AH, Thrasher TA, Kawashima N, Morris A, Preuss R, Popovic MR (2009). Postural Reactions of the Trunk Muscles to Multi-directional Perturbations in Sitting. Clin Biomech.

[20] Triolo RJ, Boggs L, Miller ME, Nemunaitis G, Nagy J, Nogan-Bailey S (2009). Implanted Electrical Stimulation of the Trunk for Seated Postural Stability and Function after Cervical Spinal Cord Injury: A Single Case Study. Arch Phys Med Rehabil.

[21] Murphy JO, Audu ML, Lombardo LM, Foglyano KM, Triolo RJ (2014). Feasibility of Closed-loop Controller for Righting Seated Posture after Spinal Cord Injury. J Rehabil Res Dev.

[22] Triolo RJ, Nogan-Bailey S, Lombard LM, Miller ME, Foglyano K, Audu ML (2013). Effects of Intramuscular Trunk Stimulation on Manual Wheelchair Propulsion Mechanics in 6 Subjects with Spinal Cord Injury. Arch Phys Med Rehabil.

[23] Ojeda M, Ding D. Temporal Parameters Estimation for Wheelchair Propulsion Using Wearable Sensors. Biomed Res Int. 2014; doi.10.1155/2014/645284. 10.1155/2014/645284 PMC4106105 25105133

[24] Garcí-Massó X, Serra P, González LM, Garcia-Casado J (2015). Identifying Physical Activity Type in Manual Wheelchair Users with Spinal Cord Injury by Means of Accelerometers. Spinal Cord.

[25] Postma K, van den Ber-Gemons HJG, Bussmann JBJ, Sluis TAR, Bergan MP, Stam HJ (1995). Validity of the Detection of Wheelchair Propulsion as Measured with an Activity Monitor in Patients with Spinal Cord Injury. J Spinal Cord Med.

[26] Hiremath SV, Ding D, Farrindon J, Vyas N, Cooper RA (2013). Physical Activity Classification Utilizing SenseWear Activity Monitor in Manual Wheelchair Users with Spinal Cord Injury. Spinal Cord.

[27] Crawford A, Armstrong K, Loparo K, Audu M, Triolo R. “Detecting Destabilizing Wheelchair Conditions for Maintaining Seated Posture. Disabil Rehabil Assist Technol. 2017. doi:10.1080/17483107.2017.1300347. 10.1080/17483107.2017.1300347 PMC5623614 28366027

[28] Bruno C (1997). Development of a Mathematical Model to Investigate the Static and Dynamic Stability of a Wheelchair System. MS Thesis, Worcester Polytechnic Institute.

[29] Majaess GG, Kirby RL, Ackroyd-Stolarz SA, Charlebois PB (1993). Influence of Seat Position on the Static and Dynamic Forward Rear Stability of Occupied Wheelchairs. Arch Phys Med Rehabil.

[30] Li WW, Shahram P. A Study of Active Shifting of Human Driver for Improving Wheelchair Tipping Stability. CiteSeerX, 2008. oai(CiteSeerX.psu):10.1.1.50.5536.

[31] Cooper RA, MacLeish M (1992). Racing Wheelchair Roll Stability While Turning: A Simple Model. J Rehabil Res Dev.

[32] Vette AH, Masani K, Popovic MR (2008). Time Delay from Muscle Activation to Torque Generation during Quiet Stance: Implications for Closed-Loop Control via FES. Biomed Tech.

[33] Nataraj R, Audu ML, Triolo RJ. Simulating the Restoration of Standing Balance at Leaning Postures with functional Neuromuscular Stimulation following Spinal Cord Injury. Med Biol Eng Comput. 2016:163–76. 10.1007/s11517-015-1377-5 PMC4775462 26324246

[34] Audu ML, Triolo RJ (2015). Intrinsic and Extrinsic Contributions to Seated Balance in the Sagittal and Coronal Planes: Implications for Trunk Control after Spinal Cord Injury. J Appl Biomech.

[35] Triolo RJ, Bailey SN, Lombardo LM, Miller ME, Foglyano K, Audu ML (2013). Effects of Intramuscular Trunk Stimulation on Manual Wheelchair Propulsion Mechanics in 6 Subjects with Spinal Cord Injury. Arch Phys Med Rehabil.

[36] Triolo RJ, Bailey SN, Miller ME, Lombardo LM, Audu ML (2013). Effects of Stimulating Hip and Trunk Muscles on Seated Stability, Posture, and Reach after Spinal Cord Injury. Arch Phys Med Rehabil.

[37] Hunt AJ, Odle BM, Lombardo LM, Audu ML, Triolo RJ (2017). Reactive Stepping with Functional Neuromuscular Stimulation in Response to Forward-directed Perturbations. J Neuroeng Rehabil..

[38] Triolo RJ, Bailey SN, Miller ME, Rohde LM, Anderson JS, Davis JA, Abbas JJ, LA DP, Forrest GP, Gater DR, Yang LJ (2012). Longitudinal Performance of a Surgically Implanted Neuroprosthesis for Lower-Extremity Exercise, Standing, and Transfers after Spinal Cord Injury. Arch Phys Med Rehabil.

[39] Fisher LE, Miller ME, Bailey SN, Davis JA, Anderson JS, Murray LR, Tyler DJ, Triolo RJ (2008). Standing after Spinal Cord Injury with Four-contact Nerve-Cuff Electrodes for Quadriceps Stimulation. IEEE Trans Neural Syst Rehabil Eng.

[40] Smith B, Tang Z, Johnson M, Pourmehdi S, Gazdik M, Buckett J, Peckham P (1998). An Externally Powered, Multichannel, Implantable Stimulator-Telemeter for Control of Paralyzed Muscle. IEEE Trans Rehab Eng.

[41] Davis JA, Triolo RJ, Uhlir JP, Bieri C, Rohde L, Lissy D (2001). Preliminary Performance of a Surgically Implanted Neuroprosthesis for Standing and Transfers – Where do we stand?. J Rehabil Res Dev.

[42] Davis JA, Triolo RJ, Uhlir JP, Bhadra N, Lissy DA, Nandurkar S, Marsolais EB (2001). Surgical Technique for Installing an 8-channel Neuroprosthesis for Standing. Clin Orthop Relat Res.

[43] Triolo RJ, Bieri C, Uhlir J, Kobetic R, Scheiner A, Marsolais EB (1996). Implanted FNS Systems for Assisted Standing and Transfers for Individuals with Cervical Spinal Cord Injuries: Clinical Case Reports. Arch Phys Med Rehabil.

[44] Memberg WD, Peckham PH, Thrope GB, Keith MW, Kicher TP (1993). An analysis of the Reliability of Percutaneous Intramuscular Electrodes in Upper Extremity FNS Applications. IEEE Trans on Rehabil Eng.

[45] Christie BP, Freeberg M, Memberg WD, Pinault GJC, Hoyen HA, Tyler DJ, Triolo RJ (2017). Long-term Stability of Stimulating Spiral Nerve Cuff Electrodes on Human Peripheral Nerves. J Neuroeng Rehabil.

[46] Steinfeld E, Danford G (1999). Enabling Environments: Measuring the Impact of Environment on Disability and Rehabilitation.

[47] Kazdin AE, Barlow DH, Hersen M (1984). Statistical Analyses for Single-Case Experimental Designs. Single Case Experimental Designs.

[48] Payton OD, Payton OD, Sullivan MS (1989). Group Experimental Designs. Research: The Validation of Clinical Practice.

[49] Currier DP, Currier DP (1990). Experimental Designs. Elements of Research in Physical Therapy.

[50] Manduchi R, Castano A, Talkukder A, Matthies L (2005). Obstacle Detection and Terrain Classification for Autonomous Off-Road Navigation. Auton Robot.

[51] Vandapel N, Huber DF, Kapuria A, Hebert M (2004). Natural Terrain Classification using 3-D Ladar Data.

[52] Hebert M, Vandapel N (2003). Terrain Classification Techniques from Ladar Data for Autonomous Navigation. Proc Collaborative Technology Alliances conference.

[53] Bellutta P, Manduchi L, Matthies K, Owens K, Rankin A (2000). Terrain Perception for Demo III. Proc IEEE Intelligent Vehicles Symposium.

[54] Castano R, Manduchi R, Fox J. Classification Experiments on Real-world Textures. Workshop on Empirical Evaluation in Computer Vision, Kauai, HI, 2001.

[55] O’Sullivan S (2003). An Empirical Evaluation of Map Building Methodologies in Mobile Robotics Using the Feature Prediction Sonar Noise Filter and Metric Grid Map Benchmarking Suite.

